# Experimental demonstration of linear and spinning Janus dipoles for polarisation- and wavelength-selective near-field coupling

**DOI:** 10.1038/s41377-019-0162-x

**Published:** 2019-06-05

**Authors:** Michela F. Picardi, Martin Neugebauer, Jörg S. Eismann, Gerd Leuchs, Peter Banzer, Francisco J. Rodríguez-Fortuño, Anatoly V. Zayats

**Affiliations:** 1 Department of Physics and London Centre for Nanotechnology, King’s College London, Strand, London, WC2R 2LS UK; 2 Max Planck Institute for the Science of Light, Staudtstr. 2, D-91058 Erlangen, Germany; 3 Institute of Optics, Information and Photonics, University Erlangen-Nuremberg, Staudtstr. 7/B2, D-91058 Erlangen, Germany

**Keywords:** Nanophotonics and plasmonics, Sub-wavelength optics

## Abstract

The electromagnetic field scattered by nano-objects contains a broad range of wavevectors and can be efficiently coupled to waveguided modes. The dominant contribution to scattering from subwavelength dielectric and plasmonic nanoparticles is determined by electric and magnetic dipolar responses. Here, we experimentally demonstrate spectral and phase selective excitation of Janus dipoles, sources with electric and magnetic dipoles oscillating out of phase, in order to control near-field interference and directional coupling to waveguides. We show that by controlling the polarisation state of the dipolar excitations and the excitation wavelength to adjust their relative contributions, directionality and coupling strength can be fully tuned. Furthermore, we introduce a novel spinning Janus dipole featuring cylindrical symmetry in the near and far field, which results in either omnidirectional coupling or noncoupling. Controlling the propagation of guided light waves via fast and robust near-field interference between polarisation components of a source is required in many applications in nanophotonics and quantum optics.

Scattered fields from plasmonic and dielectric nanostructures contain a broad range of wavevectors^1^, which makes them suitable for efficient coupling to waveguided modes. Such nanostructures underpin applications in photonic data manipulation, quantum technologies and precision metrology. Nanoparticle scattering is often dominated by lowest-order multipoles, electric and magnetic dipoles, whose near- and far-field interference can be controlled at ultrafast speeds by tuning the amplitudes and phases between dipolar components^2–4^. For example, spin-momentum locking of guided light that is excited by circularly polarised dipoles^5–10^ has led to numerous applications in quantum optics^11–13^ and optical manipulation^14–16^. Recently, a dipolar source that exhibits a face-dependent coupling behaviour was theoretically predicted, the “Janus dipole”, which is composed of out-of-phase electric and magnetic dipoles^17^. Here, we experimentally demonstrate the excitation of such a Janus dipole using a silicon nanoparticle and measure its characteristic near-field angular spectral signature. Furthermore, we introduce a *spinning* Janus dipole that features cylindrical symmetry, which provides either omnidirectional coupling or noncoupling.

Previous experimental demonstrations of near-field directional coupling of dipolar sources have been realised with circularly polarised dipoles. While circularly polarised electric dipoles, which are comprised of two orthogonal linear electric dipoles that oscillate with a phase difference of ± *π*/2, excite unidirectionally *p*-polarised waveguide modes, circular magnetic dipoles can be used to excite *s*-polarised modes^18^. By superimposing electric and magnetic dipole contributions, additional degrees of freedom in engineering directionality can be harnessed via their interference^19–21^. For example, the well-known Huygens dipole is a combination of orthogonally oriented, in-phase electric **p** and magnetic **m** dipoles that satisfy Kerker’s scattering condition, which is expressed as *p* = *m/c*, where *c* denotes the speed of light. This source has been experimentally demonstrated to exhibit directionality in the far-field^22–24^ and is employed in reflectionless dielectric metasurfaces^25,26^. However, when the electric and magnetic dipoles are perpendicular to each other, as in the Huygens dipole, but ± *π*/2 out of phase, the resulting source is the so-called Janus dipole, which has only recently been predicted theoretically^17^. The Janus dipole earns its name from the dependence of its observed behaviour on which side of this source faces a nearby waveguide. One face will couple to guided modes, while the opposite one will exhibit a complete absence of coupling. This behaviour is reversed by flipping the polarisation of the dipole by switching between the two faces. Unlike circular dipoles, whose directionality can be switched experimentally by changing the polarisation of the plane wave illuminating the nanoparticle, the Janus and Huygens dipoles’ directionalities cannot be controlled in this way using spherical, isotropic nanoparticles as scatterers^17^. Janus, Huygens, and circularly polarised dipoles were identified as the three elemental dipolar sources for directional mode excitation in planar geometries^17^. All these sources are based on the same fundamental principles of near-field interference and provide broadband operation.

Here, we experimentally demonstrate wavelength-selective excitation of Janus dipole sources, along with their directional coupling properties, by tailoring the near-field interference between the electric and magnetic dipole moments that are induced in dielectric nanoparticles. We demonstrate that by tuning the polarisation state of the excited dipoles and the excitation wavelength to adjust their relative contributions, various dipolar sources can be realised, including the linear Janus dipole. In addition, we discuss and experimentally demonstrate the possibility of realising omnidirectional coupling or noncoupling with a novel spinning Janus dipole.

A dipolar source can be realised experimentally by illuminating any small nanostructure which scatters in the lowest-order Mie regime^8,22^. Simultaneous electric and magnetic dipolar excitations will be realised if both its electric and magnetic polarisabilities are nonzero^23,25,27,28^. Plane-wave illumination conveniently provides orthogonal electric **E** and magnetic **H** fields, which match the **p** and **m** dipole moment directions that are required for the linear Janus dipole. However, the orthogonal fields of plane waves are always in phase. To obtain a Janus source for which the electric and magnetic dipole moments are phase shifted, we can exploit the intrinsic wavelength-dependent phase difference between the electric and magnetic polarisabilities of the particle^8^. When this phase difference equals ± *π*/2 and the amplitudes of the electric and magnetic dipole moments are comparable, a Janus dipole is realised (Fig. 1a).

**Fig. 1:**
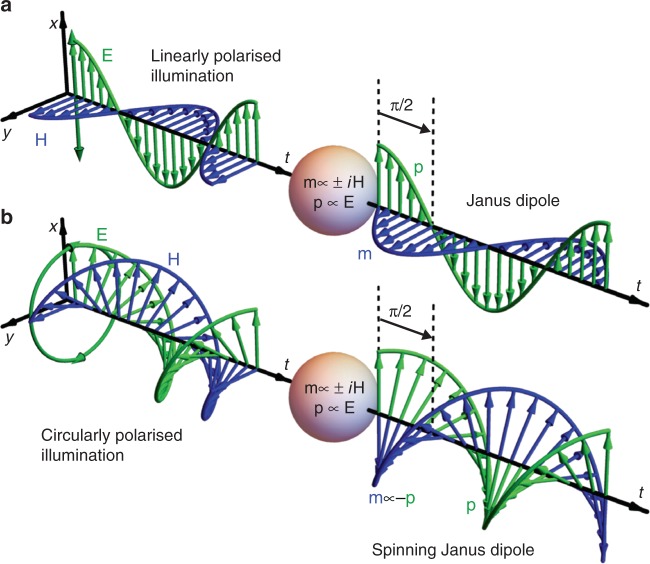
Linear and spinning Janus dipoles. A nanoparticle whose electric and magnetic polarisabilities have a fixed phase difference at a specified wavelength scatters light like a dipolar source with electric and magnetic dipole moments that feature a phase difference determined by the intrinsic polarisabilities. Left: incident **E** and **H** fields as functions of time. Right: dipole moments **p** and **m** of the nanoparticle as functions of time. When the phase difference between the two polarisabilities is *π*/2, **a** the nanoparticle under linearly polarised plane wave illumination will scatter like a linear Janus dipole. **b** The same nanoparticle under circularly polarised plane wave illumination will scatter like a spinning Janus dipole (the electric and magnetic fields rotate in the same way and are oriented antiparallel at all times). An additional global phase-delay between the excitation fields and the resulting dipoles is omitted from the sketch

High-index dielectric nanoparticles, such as silicon particles, are suitable for this purpose since they possess both electric and magnetic Mie resonances^2^. For small enough nanoparticles, higher order multipole resonances can be safely neglected in the visible spectrum^27^. By tuning the wavelength and polarisation of the illumination, we can select the amplitudes, directions, and phase difference of the electric and magnetic dipole moments in the nanoparticle; hence, it is the ideal candidate for experimentally realising a Janus dipole source.

The unique coupling behaviour of a Janus dipole with a waveguide is closely related to the reactive power of the evanescent tails in the mode that is being excited^17^. The reactive power is the vector Im{E^*^ × **H**}, namely, the imaginary part of the Poynting vector. The coupling or noncoupling behaviour of the Janus dipole depends on whether the corresponding vector quantity, Im{p^*^ × **m**}, of the source is pointing in the same or opposite direction as the reactive power of the mode, which gives rise to its two faces. The direction of the reactive power of an evanescent wave depends on its polarisation^29^: *s*-polarised waves (also called transverse electric waves, with no electric field component in the direction of propagation) have a reactive power that points in the direction of the evanescent decay, while the reactive power of *p*-polarised modes (transverse magnetic modes) is opposite the direction of decay. Therefore, the definitions of coupling and noncoupling faces of a Janus dipole depend on the polarisation of the excited mode. In this work, we experimentally generate both a linear and a spinning Janus dipole with Im{p^*^ × **m**} pointing towards a nearby medium of higher optical density (glass with a refractive index of 1.5), thereby resulting in preferred *p*-polarised and strongly suppressed *s*-polarised evanescent coupling between the dipole and the medium. We observe this behaviour by measuring the angular spectrum of the sources in the glass half-space (similar to the measurements in^8^; see SM for details).

Because of the small distance between the dipolar source and the substrate, both the propagating and evanescent wavevector components of the source can couple into propagating waves inside the glass, which can be measured. We are interested in the emission that corresponds to evanescent fields in free-space, which is responsible for the near-field directionality of the Janus dipole, with $\frac{{k_t}}{{k_0}} > 1$, where *k*_*t*_ is the transverse wavevector that is perpendicular to the optical axis (*z*) and *k*_0_ is the wavenumber in free space. These fields, which are evanescent in free space and become propagating in optically denser media, are sometimes referred to as “forbidden light”^30^. Although the amplitude of the measured spectrum will be a modified version of the near-field spectrum of the isolated Janus source in free space, the difference can be calculated via a multiplicative transfer function that accounts for the polar-angle dependence of the Fresnel transmission coefficients through the high-index substrate interface. Therefore, any zeroes in the angular spectrum of the free-space source will also be present in the measured angular spectra, which follows directly from the conservation of transverse momentum. The arrangement of zeroes in the spectra are a clear signature of a Janus dipole (see SM). For instance, a Janus dipole with $\frac{{p_x}}{{m_y}} = - \frac{{iR}}{c}$, where *R* is a normalised measure of the ratio of electric to magnetic components, shows zero amplitude for the *s*-polarised evanescent components with $k_t = k_0\sqrt {R^2 + 1}$ on its noncoupling side (z > 0), which is due to the destructive interference between the electric and magnetic dipole fields after their superposition. Then, a Janus dipole that satisfies *p*_*x*_/*m*_*y*_ = −*i/c* would lead to a ring of zero intensity at the transverse *k*-vector, that corresponds to $k_t = \sqrt 2 k_0$^17^. However, this would exceed the angular range of our experimental setup. Thus, our ideal Janus dipole condition is *p*_*x*_/*m*_*y*_ = −*i*0.75/*c*, which is optimised for *k*_*t*_ = 1.25 *k*_0_.

For our experiment, we place an individual silicon nanosphere (diameter approximately 176 nm) on a glass substrate (see Fig. 2a) on the optical axis of a linearly *x*-polarised Gaussian beam (focused with an effective NA of 0.5) that is used for excitation. For this configuration, due to the linearly polarised illumination, we excite an *x*-polarised electric dipole, namely, *p*_*x*_, and a *y*-polarised magnetic dipole, *m*_*y*_. Then, we can control the amplitudes of and the relative phase between the two dipole moments by selecting the wavelength of the excitation field. Between the magnetic and electric dipole resonances (Fig. 2b,) we expect two wavelengths for which the relative phase between *m*_*y*_ and *p*_*x*_ is close to *π/*2 (the Janus dipole condition) with both dipole amplitudes being of comparable strength. Due to the presence of the substrate, these will differ slightly from those that are predicted by free-space Mie theory. Nonetheless, the free-space scattering cross-section provides a range within which the wavelength can be fine-tuned experimentally. Then, we measure the intensity distribution in the back focal plane (BFP) of an oil immersion objective (NA = 1.3) that is placed below the glass substrate to capture the near- and far-field parts of the angular spectrum of the Janus dipole for 0.6 < *k*_*t*_/*k*_0_ < 1.3. The angular range below an NA of 0.6 is also collected but discarded because it contains the transmitted input beam. The collected spectrum is analysed with a linear polariser to retrieve its *s*- and *p*-polarisation components.

**Fig. 2:**
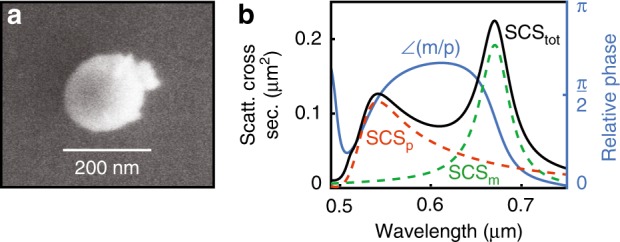
Nanoparticle properties. **a** A SEM image of a spherical Si particle with core radius of r ≈ 84 nm and an oxide shell thickness of s ≈ 4 nm. **b** The total scattering cross-section (solid black line) and the relative phase between the resonances (solid blue line) according to calculations that use Mie theory for the nanoparticle in (**a**). The dashed orange and green lines represent the electric and magnetic dipole scattering cross-sections, which are proportional to $\left| {\mathbf{p}} \right|^2$ and $\left( {\left| {\mathbf{m}} \right|/c} \right)^2$, respectively

Figure 3 shows the results of the measurements, together with the calculated dipoles, that were obtained for three wavelengths of illumination: (a) *λ* = 520 nm, (b) *λ* = 640 nm, and (c) *λ* = 700 nm. We observe a striking agreement between the measured data and the results of the numerical calculations. At *λ* = 640 nm, we are very close to the linear Janus dipole condition (Fig. 3b), for which the electric and magnetic dipole moments have a relative phase that is close to *π*/2 and an amplitude ratio of $\left| {p_x/m_y} \right| \approx 0.75/c$. A ring of zero amplitude outside the light cone (which corresponds to near fields) in the *s*-polarised angular spectrum of Fig. 3b is a clear signature of the noncoupling face of the Janus dipole. Our measurements reveal that the amplitude of its angular spectrum is zero for a circle with transverse wavevector *k*_*t*_ = 1.25 *k*_0_ as analytically expected. Hence, if placed near a waveguide that is supporting an *s*-polarised mode with this or a similar propagation constant, this source will not be able to excite it in *any* direction due to a momentum mismatch. In contrast, the *p*-polarised component is non-zero everywhere except for the *k*_*x*_ = 0 line. The source will excite *p*-polarised modes in all directions except for the ± *y*-direction. These are trivial zeroes because they result from a polarisation mismatch: the dipole has components *p*_*x*_ and *m*_*y*_; however, *p*-polarised modes that are propagating parallel to the *y*-direction do not feature the corresponding *E*_*x*_ and *H*_*y*_ field components for coupling. Reversing the Janus dipole reverses the coupling/noncoupling behaviour: the *s*-polarised component becomes non-zero everywhere and, hence, coupling, while the *p*-polarised component features the noncoupling spectrum. To reverse the Janus dipole, one must change the relative phase between **p** and **m** by 180°. One way of reversing a Janus dipole that is induced via plane-wave illumination would be to invert the illumination direction. Due to the relation (**E** × **H**)∝**k** in plane waves, changing the direction of **k** corresponds to a sign change of either **E** or **H**, but not of both simultaneously. Thus, polarisation that was coupling would become non-coupling and vice versa.

**Fig. 3:**
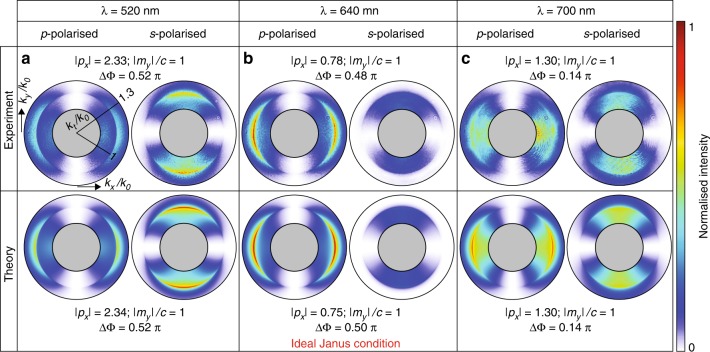
Near-field scattering of dipolar sources and their spectral dependence. The measured (top) and calculated (bottom) BFP intensities of *s*- and *p*-polarised near-field angular distributions for **a** *λ* = 520 nm, **b** *λ* = 640 nm, and **c** *λ* = 700 nm that correspond to various relative contributions of electric and magnetic dipoles and their relative phases. The sets of *s*- and *p*-polarised intensity distributions are normalised to their common maximum value. For *λ* = 640 nm, the Janus condition ($\left| {p_x/m_y} \right| = 0.75/c$ and ΔΦ = arg(*m*_*y*_/*p*_*x*_)=π/2) is theoretically satisfied. The *s*-polarised component of the light that is scattered by the Janus dipole presents a full ring of zero intensity at the locations $k_x^2 + k_y^2 = k_t^2 = k_0^2\left( {R^2 + 1} \right),$ which correspond to the noncoupling condition for the specified *amplitude* of the wavevector *k*_*t*_ in *any* direction. In (a) and (c), the dipole moments and the phase difference that are used in the theoretical plots are the same as the experimental values, while in (b) the simulation shows the ideal Janus dipole, with $\left| {p_x/m_y} \right| = 0.75/c$ and ΔΦ = *π*/2

Figure 3a, c show the angular spectra that were obtained at two other wavelengths, namely, 520 nm and 700 nm, respectively, for which the Janus condition is not fulfilled and, therefore, the aforementioned feature of noncoupling cannot be realised. From the measured angular spectra, we determine the corresponding dipole moments that are induced in the nanoparticle^3,21^. For this purpose, we performed a nonlinear least-square fit of theoretically calculated far fields to our measured angular spectra. A more detailed description of the retrieval of the dipole moments is provided in the supplementary material. At *λ* = 520 nm, the amplitude of the magnetic dipole moment is substantially smaller than that of the electric dipole moment $\left( {\left| {p_x/m_y} \right| \approx 2.3/c} \right)$. Hence, even if the phase between them is close to *π*/2, the destructive interference condition is satisfied at transverse wavevectors, namely, *k*_*t*_/*k*_0_ ≫ NA, which well exceeds the available numerical aperture in the experiment. In the measured angular region, the electric dipole behaviour will be dominant and the nanoparticle will scatter like an electric dipole that is polarised along *x*. In contrast, for *λ* = 700 nm, the amplitudes of the two dipole moments are comparable, namely, $\left| {p_x/m_y} \right| \approx 1.3/c$; however, the phase between the two is almost zero: ΔΦ = 0.14rad. To quantitatively compare all three excited dipoles and their polarisation-dependent coupling to evanescent waves, we further investigate the measured angular spectra in the region above the critical angle (*k*_*t*_/*k*_0_ < 1.3). The ratios between the integrated *p*- and *s*-polarised intensities are 1.5:1 (520 nm), 11.5:1 (640 nm) and 1.8:1 (700 nm). These results highlight again the noncoupling nature of the *s*-polarised light of the Janus dipole. The overall scattering efficiency into the super-critical angular regime of our system can be defined as the ratio between the power of the light that is scattered into this region and the power of the angular spectrum of the incoming beam of light. For the three wavelengths, we acquired similar values of ~1.6/%, ~1.7/%, and ~1.4/%. In principle, these numbers can be increased by using tightly focused beams with a higher effective NA for excitation.

Due to the linearly polarised illumination, **p** and **m** are always pointing along *x* and *y*, respectively (Fig. 1a), which is the reason for the lines of zero amplitude (*k*_*y*_ = 0 for *s*-polarised light and *k*_*x*_ = 0 for *p*-polarised light) that are clearly visible in all angular spectra in Fig. 3. These zeroes are caused by a polarisation mismatch between the dipole and the modes, as described above, rather than the destructive interference between **p** and **m** that is characteristic of the Janus dipole.

These trivial lines of zero amplitude in the spectra can be removed via illumination with circularly polarised light, which should result in the excitation of **p** and **m** with the same time-dependence as the illuminating **E** and **H** fields, but with a phase delay that corresponds to *π*/2, as a direct consequence of the particle’s response (Fig. 1b.) This illumination induces electric and magnetic dipoles that are circularly polarised and spin together in the *xy* plane but are oriented antiparallel at all times, such that **p** = (1,−*i*,0) and **m**/*c* = −**p**/*R*. This source constitutes a novel “spinning” Janus dipole, with a non-zero associated vector Im{p^*^ × **m**} that is directed along + *z* towards the substrate, as required for noncoupling to *s*-polarised modes. The angular spectrum intensity of this dipole is rotationally symmetric, as it exhibits no polarisation mismatch to modes in any direction. The stark contrast between *p*-polarised coupling and *s*-polarised noncoupling in the evanescent region is even clearer in the experiment. The full ring of zeroes is caused purely by the interference of **p** and **m**, which is characteristic of the Janus dipole (Fig. 4). The source couples to *p*-polarised evanescent waves in all directions, while it does not couple to *s*-polarised evanescent waves with a fixed *k*_*t*_ > *k*_0_ at any angle. This behaviour would be reversed for an opposite sign of the *π*/2 phase difference between the electric and magnetic polarisabilities that are induced by the nanoparticle. In this case, the induced electric and magnetic dipoles are spinning parallel to each other and the source is noncoupling for *p*-polarised modes.

**Fig. 4:**
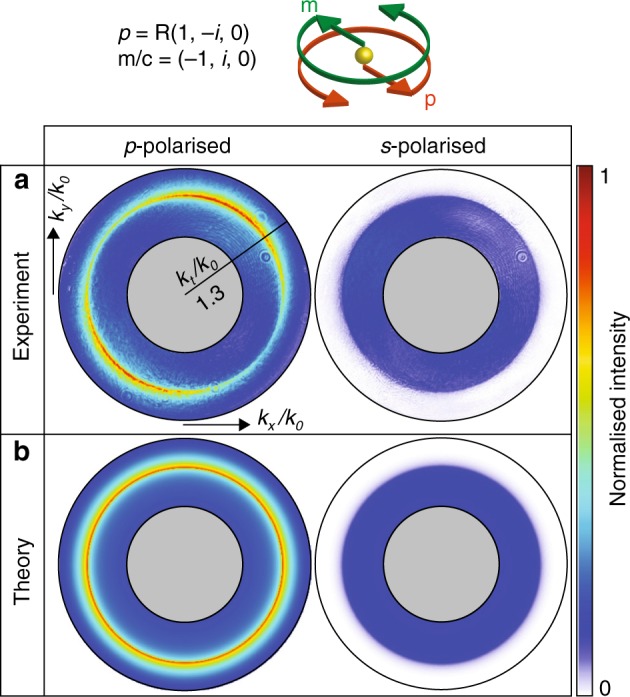
Spinning Janus dipole. Measured (**a**) and calculated (**b**) BFP intensities of the *p*- and *s*- polarised scattering from a spinning Janus dipole. The experimental and theoretical distributions are normalised to their common maximum value. The retrieved experimental dipole moments are ***p*** = (0.61−0.11*i*,−0.01−0.75*i*,0) and **m**/*c* = (−1,0.30 + 0.84*i*,0), while the theoretical dipole moments are ***p*** = 0.75(1,−*i*,0) and **m**/*c* = (−1,*i*,0). The slight asymmetry in the experimental results is attributed to the experimental dipole moments not matching exactly the ideal ones for which the spinning Janus dipole is cylindrically symmetric

In conclusion, the experimental measurement of the Janus dipole supports the theoretical predictions of a source with a polarisation-dependent omnidirectional absence of coupling to evanescent waves, which adds to the already widely used circular and Huygens dipoles as an extra source with a polarisation-controllable near field. The striking agreement between the dipoles that are obtained from the scattering from the silicon nanoparticle and the theoretical point sources demonstrates the effectiveness of the utilised dipolar approximation. Moreover, the sensitivity of the response to the illumination parameters leaves room for applications in which a different phase and amplitude ratio between the dipole components may be required, including guided modes with different transverse wavevectors, which can be matched to the source by properly tuning the dipole components. This experimental demonstration highlights the feasibility of the Janus source, thereby paving the way towards novel applications in nanophotonics, quantum information and plasmonics, which might include the Janus dipole and its spinning version.

## Supplementary information

Supplementary information
