# Supplementary material for: Experimental demonstration of linear and spinning Janus dipoles for polarisation- and wavelength-selective near-field coupling
Source: Light Sci Appl. 2019 Jun 5;8:52. doi: 10.1038/s41377-019-0162-x (PMC6548777; doi:10.1038/s41377-019-0162-x)
Supplement: Supplementary file 1 — Supplementary information [file 41377_2019_162_MOESM1_ESM.pdf]

Supplementary information
Experimental demonstration of linear and spinning Janus dipoles for polarisation- and wavelength-selective near-field coupling

Michela F. Picardi,[1,*] Martin Neugebauer,[2,3,*] Jörg S. Eismann,[2,3,*] Gerd Leuchs,[2,3] Peter Banzer,[2,3] Francisco J. Rodríguez-Fortuño,[1] and Anatoly V. Zayats[1]

[1]Department of Physics and London Centre for Nanotechnology, King's College London, Strand, London, WC2R 2LS, United Kingdom
[2]Max Planck Institute for the Science of Light, Staudtstr. 2, D-91058 Erlangen, Germany
[3]Institute of Optics, Information and Photonics, University Erlangen-Nuremberg, Staudtstr. 7/B2, D-91058 Erlangen, Germany

The angular spectrum

In order to characterise the fields radiated by a Janus dipole and its behaviour when coupling to nearby waveguides, we can use the angular spectrum representation. This is a convenient approach to predict and optimise directionality in near field coupling [1]. The fields from the source are written as a sum of all its spectral components, both propagating plane waves and non-propagating evanescent waves, each corresponding to a transverse wavevector in the plane $\mathbf{k}_t = (k_x, k_y)$ and each with a given amplitude and phase. In free space, the radiation of a source located at $\mathbf{r} = 0$ is simply the superposition of all these contributions:

$$\mathbf{E}(x, y, z) = \iint \mathbf{E}(\mathbf{k}_t)|_{z=0} e^{i(k_x x + k_y y + k_z |z|)} dk_x dk_y, \tag{S1}$$

where $k_z = \sqrt{k^2 - k_x^2 - k_y^2}$ taking always the positive root, and $k = \omega/c$ is the wavenumber of free space.

This corresponds to the fields of the source in free space. However, whenever we wish to study the coupling of a source to a waveguide, thanks to the conservation of momentum parallel to interfaces, the only relevant spectral components are the ones whose transverse wavevector matches those of the modes supported by the waveguide. In other words, what matters in the coupling is the similarity between the k-spaces spanned by the source fields and by the waveguide mode fields. With these assumptions it is then clear that in order for a mode to be excited by the source, the source must have the spectral component with the same wavevector of the mode and, therefore, to achieve non-coupling between a source and a guided mode it is sufficient to remove that spectral component from the source. Then, if a source "lacks" some spectral components it will not be possible to excite the waveguide modes with the corresponding wavevector, independently of the distance between the source and the waveguide.

The key to achieve near-field destructive interference for a given mode is, therefore, to engineer a source "lacking" the spectral component corresponding to the mode. To achieve the omnidirectional noncoupling of the s-polarised modes on a planar waveguide with propagation constant k_m, a Janus dipole can be used, with the amplitude of all the spectral components of the dipole source being zero on a circumference of radius $|\mathbf{k}_t| = k_m$. Therefore, we can focus our analysis on the free-space angular spectrum of the source and this will straightforwardly determine directional excitation of guided modes if the source was placed in close proximity of a waveguiding structure.

In a most general case, the free-space electric field angular spectrum amplitudes of any dipolar source, generated by a superposition of an electric and a magnetic dipole, can be written as (see [1] and supplementary information of [2]):

$$\mathbf{E}(\mathbf{k}_t)|_{z=0} = \frac{ik^2}{8\pi^2\varepsilon} \frac{1}{k_z} \left[(\mathbf{v} \cdot \hat{\mathbf{e}}_s)\, \hat{\mathbf{e}}_s + \left(\mathbf{v} \cdot \hat{\mathbf{e}}_p^{\pm}\right) \hat{\mathbf{e}}_p^{\pm} \right], \tag{S2}$$

with $\mathbf{v} = \mathbf{p} - \hat{\mathbf{k}} \times (\mathbf{m}/c)$, $\hat{\mathbf{k}} = \frac{\mathbf{k}}{k} = (k_x, k_y, \pm k_z)/k$ is the normalised wavevector, and $\hat{\mathbf{e}}_s = \frac{1}{k_t}(-k_y, k_x, 0)$ and $\hat{\mathbf{e}}_p^{\pm} = \frac{1}{k}\left(\frac{\pm k_z k_x}{k_t}, \frac{\pm k_z k_y}{k_t}, -k_t\right)$ are the unit vectors relative to s- and p-polarisations respectively. Notice that the terms $\hat{\mathbf{e}}_s$, $\hat{\mathbf{e}}_p^{\pm}$ and $\mathbf{v}$ are all functions of $\mathbf{k}_t$, and the last two have a sign choice in k_z, so the angular spectrum Eq. (S2) depends on whether we are calculating the field at $z > 0$ or $z < 0$ respectively. This difference is crucial as it is the origin of the Janus dipole having two "faces". Also notice that Eq. (S2) decomposes the angular spectrum vector into a superposition of two complex amplitudes scaling the two unit vectors corresponding to s- and p-polarisations.

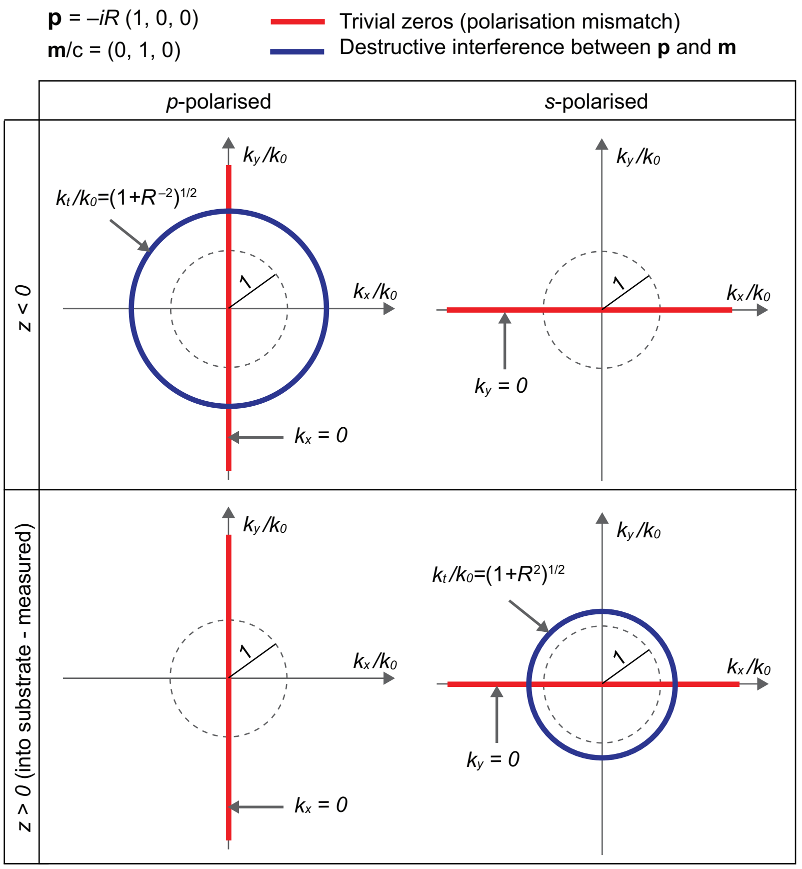

FIG. S1: Schematic representation of the zeros in the angular spectrum of a linear Janus dipole, for both polarisations, and for propagation both above and below the source. The zeros in the angular spectrum will be preserved even after transmission through the planar substrate, due to conservation of transverse momentum.

In order to analyse the near-field non-coupling behaviour of a source, we can plot the locus of values of $\mathbf{k}_t$ for which $\mathbf{E}(\mathbf{k}_t) = 0$ for each of the two polarisation components. Fig. S1 depicts these locations of the zeros in the angular spectrum of a linear Janus dipolar source with dipole moments $p_x/m_y = -iR/c$ and $R > 0$ following Eq. (S2). The figure plots the p-polarised and s-polarised spectral planes of the source on its two “faces”, corresponding to $z > 0$ and $z < 0$. The angular spectra for $z > 0$ and $z < 0$ swap places if $R < 0$, corresponding to “flipping the face” of the Janus dipole. For the spinning Janus dipole described in the main text, $\mathbf{p} = (1, -i, 0)$ and $\mathbf{m}/c = -\mathbf{p}/R$, the lines of trivial zeros disappear from the spectrum, while the circles of destructive interference between $\mathbf{p}$ and $\mathbf{m}$ remain unaltered.

Experimental setup

A simplified version of the experimental arrangement utilised for measuring the angular spectra of the individual dipole moment combinations is sketched in Fig. S2(a). An incoming monochromatic collimated Gaussian beam of light passes through a linear polariser (transmission axis defined as x-axis, power of the transmitted beam of the order of $1\,\mu$W) and is focused by a first microscope objective with a numerical aperture (NA) of 0.6 (aperture filling factor ≈ 0.8). The focused beam impinges onto a silicon nanosphere (core radius $r = 84\,$nm and estimated oxide shell thickness $s = 4\,$nm, see Fig.2 of the manuscript) sitting on a glass-substrate, which is attached to a 3D-piezo-stage. The particle is placed on the optical axis of the beam, which results in the excitation of an x-oriented electric and a y-oriented magnetic dipole moment [Fig. S2(b)]. A second microscope objective (immersion-type with NA = 1.3, index-matched to the glass substrate) is attached from below, collecting the transmitted beam and the light scattered by the particle. The polarisation of the transmitted light is analyzed using a second rotatable polariser. Finally, the back focal plane of the second objective is imaged onto a CCD-camera.

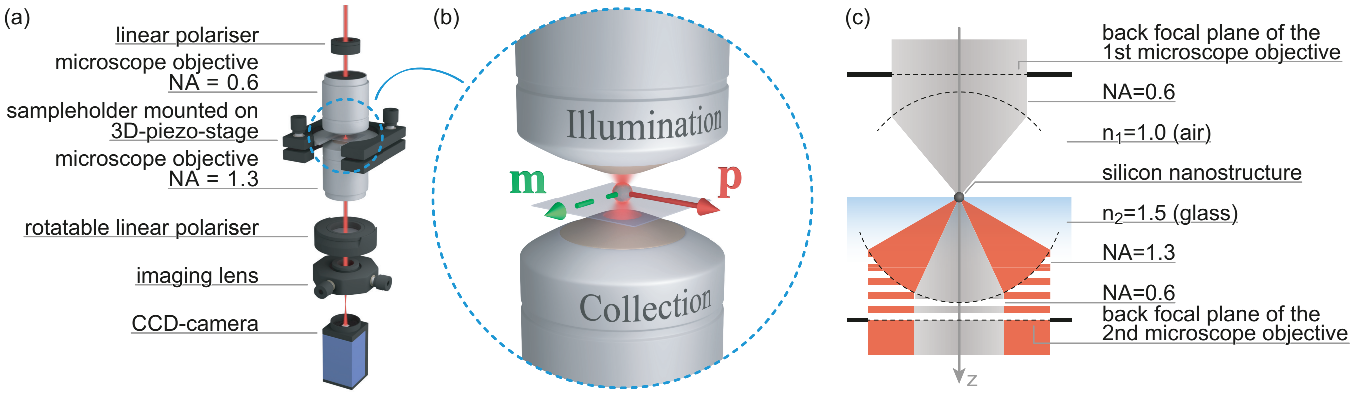

FIG. S2: Experimental concept and setup. (a) Simplified sketch of the main components of the experimental setup. (b) Magnified central part. The electric and magnetic dipole moments ($\mathbf{p}$ and $\mathbf{m}$) induced in the silicon nano sphere are indicated as red and green arrows, respectively. (c) Excitation and detection geometry.

In order to adapt the setup for the excitation of a spinning Janus dipole moment, an additional $\lambda/4$-retarder needs to be introduced between the linear polariser and the first objective, converting the linear polarisation of the incoming beam into circularly polarised light.

Since the first objective used for focusing has a smaller NA than the second objective used for collecting the light, in a certain angular range it is possible to detect the scattered light only. The principle is similar to a dark field microscope [Fig. S2(c)]. The incoming and transmitted beam corresponding to an NA of 0.6 is depicted in gray. The red circular sectors indicate the angular range in which we detect only the light scattered by the particle. Within the angular range defined by $1.0 \geq k_t/k_0 \geq 0.6$, we detect the scattered light associated with the propagating part of the angular spectrum above the glass interface. In the range $1.3 \geq k_t/k_0 > 1.0$, we detect the initially evanescent part of the angular spectrum. The upper limit of 1.3 represents the NA of the collecting objective.

Fitting of dipole moments

In the manuscript, we plot experimentally measured and theoretically calculated polarisation resolved back focal plane intensity distributions for linear and spinning Janus dipoles [see Figs. 3(b) and 4], respectively. For the theoretical distributions, we thereby assume ideal dipole moments, with a relative phase of $\pi/2$ and an amplitude ratio of $|\mathbf{p}|/|\mathbf{m}| = 0.75/c$. In addition, we provide the experimental dipole moment ratios, which we acquired by fitting theoretical s- and p-polarised intensity patterns to the experimental data, obtaining $(p_x, m_y) \propto (0.77, 0.07 + 1.00i)$ for the linear *Janus* dipole and $(p_x, p_y, m_x, m_y) \propto (1, 0.19 - 1.19i, -1.58 - 0.27i, 0.25 + 1.41i)$ for the spinning *Janus* dipole. This is done by utilizing a nonlinear least square fit where we used the amplitudes and phases of the Cartesian dipole moments $(p_x, p_y, p_z, m_x, m_y, m_z)$ of the calculated far fields as free parameters. Dipole moments which are expected to be of negligible strength are excluded from the fit and their corresponding amplitudes are set to zero, e.g. for the linear Janus dipole excited on-axis with a linearly x-polarised beam, we set p_y, p_z, m_x and m_z to zero. For the sake of completeness, we show the fitted back focal plane intensity distributions in Figs. S3(a) and (b). The first and third rows represent the experimental and the ideal theoretical results as depicted in the manuscript. The second row corresponds to the fitted distributions (not shown in the manuscript).

For the linear Janus dipole, experiment, fit, and ideal theory are all in very good agreement. However, in case of the spinning Janus dipole we see a mismatch between the ideal theory and the experimental data. In the experiment we observe an asymmetric scattering pattern, whereas the theoretical calculations predict a perfectly symmetric angular spectrum. The fitted distributions, however, show a very good overlap with the experimental data. This indicates that the minor aberrations of the experimental data can be explained by an amplitude and phase mismatch of the individual components of the experimental dipole moment with respect to the ideal dipole moment.

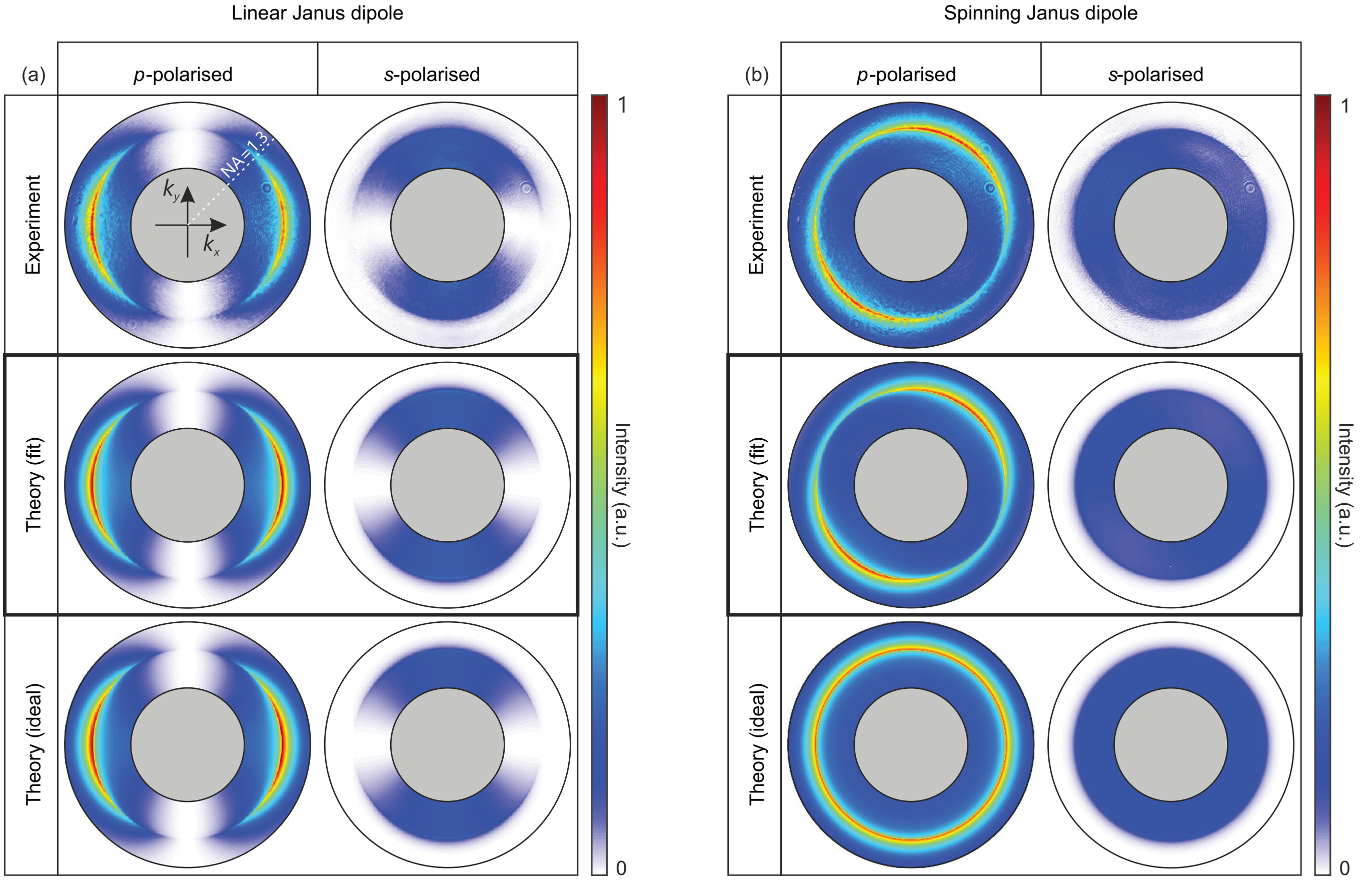

FIG. S3: Linear and spinning Janus dipoles. The first and third rows in (a) and (b) depict the measured and the theoretical (ideal) p- and s-polarised intensities of a linear and a circular polarised Janus dipole, respectively. The results are shown in Figs.3 and 4 of the main text. The back focal plane intensity distributions in the second rows depict the intensity distributions for the dipole moments fitted to the experimental observations. Each set of p- and s-polarised intensities is normalised to its common maximum value.

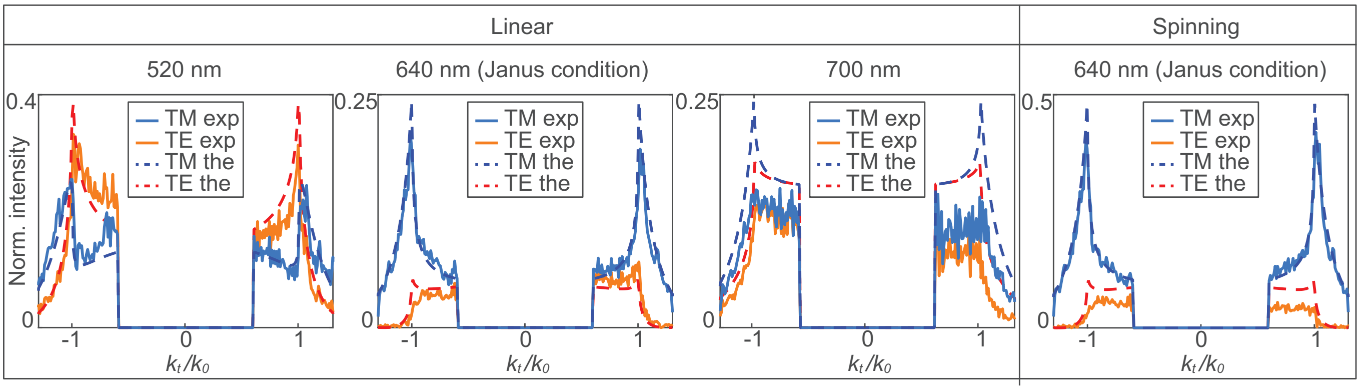

FIG. S4: Scattered intensity profiles. The intensity profiles obtained from the cross-sections of the experimental (solid lines) and theoretical (dashed lines) intensity maps of Figs. 3 and 4 in the main text. The cross-sections are performed along the line $k_x = k_y$ to avoid measuring any of the trivial zeros along the k_x and k_y axes.

* These authors contributed equally to the work.

[1] M. F. Picardi, A. Manjavacas, A. V. Zayats, and F. J. Rodríguez-Fortuño, Physical Review B **95**, 245416 (2017).
[2] M. F. Picardi, A. V. Zayats, and F. J. Rodríguez-Fortuño, Physical Review Letters **120**, 117402 (2018).
